# Impact of Neck Dissection in Head and Neck Squamous Cell Carcinomas of Unknown Primary

**DOI:** 10.3390/cancers13102416

**Published:** 2021-05-17

**Authors:** Yazan Abu-Shama, Julia Salleron, Florent Carsuzaa, Xu-Shan Sun, Carole Pflumio, Idriss Troussier, Claire Petit, Matthieu Caubet, Arnaud Beddok, Valentin Calugaru, Stephanie Servagi-Vernat, Joël Castelli, Jessica Miroir, Marco Krengli, Paul Giraud, Edouard Romano, Jonathan Khalifa, Mélanie Doré, Nicolas Blanchard, Alexandre Coutte, Charles Dupin, Shakeel Sumodhee, Yungan Tao, Vincent Roth, Lionel Geoffrois, Bruno Toussaint, Duc Trung Nguyen, Jean-Christophe Faivre, Juliette Thariat

**Affiliations:** 1 Department of Oto-Rhino-Laryngology—Head and Neck Surgery, Centre Hospitalier Régional Universitaire, Vandœuvre-Lès-Nancy, 54519 Nancy, France; yazan.abushama@gmail.com (Y.A.-S.); b.toussaint@chru-nancy.fr (B.T.); dtrungnguyen02@gmail.com (D.T.N.); 2 Department of Biostatistics and Data Management, Institut de Cancérologie de Lorraine, 54519 Nancy, France; j.salleron@nancy.unicancer.fr; 3 Department of Oto-Rhino-Laryngology—Head and Neck Surgery, Centre Hospitalier Régional Universitaire, 86021 Poitiers, France; florent.carsuzaa@gmail.com; 4 Department of Radiotherapy, CHRU de Besançon—Montbéliard, 25000 Besançon, France; Xu-Shan.SUN@hnfc.fr (X.-S.S.); matthieu.caubet@gmail.com (M.C.); 5 Department of Oncology, Institut de Cancérologie de Lorraine, 54519 Nancy, France; pflumiocarole@gmail.com (C.P.); l.geoffrois@nancy.unicancer.fr (L.G.); 6 Centre des Hautes Energies, Department of Radiotherapy, 06000 Nice, France; idrisstroussier@hotmail.com (I.T.); Yungan.TAO@gustaveroussy.fr (Y.T.); 7 Department of Radiotherapy, Institut Gustave Roussy, 94805 Villejuif, France; claire.petit@hotmail.fr; 8 Department of Radiotherapy, Institut Curie, 75005 Paris, France; a.beddok@gmail.com (A.B.); valentin.calugaru@curie.fr (V.C.); 9 Department of Radiotherapy, Institut Jean Godinot, 51100 Reims, France; stephanie.servagi@gmail.com; 10 Department of Radiotherapy, Institut Eugène Marquis, 35000 Rennes, France; j.castelli@rennes.unicancer.fr; 11 Centre Jean Perrin, Department of Radiotherapy, 63011 Clermont-Ferrand, France; miroirjessica@hotmail.fr; 12 Department of Radiotherapy, University of Piemonte Orientale, 27100 Pavia, Italy; marco.krengli@med.uniupo.it; 13 Department of Radiotherapy, Centre Hospitalier Universitaire Tenon—Assistance Publique–Hôpitaux de Paris, 75020 Paris, France; p-giraud@outlook.fr (P.G.); ed-romano@hotmail.fr (E.R.); 14 Department of Radiotherapy, Institut Universitaire du Cancer, 31100 Toulouse, France; jonathan.khalifa@hotmail.fr; 15 Department of Radiotherapy, Institut de Cancérologie de l’Ouest, 44800 Nantes, France; melanie.dore@ico.unicancer.fr; 16 Department of Radiotherapy, Clinique les Dentellières, 59300 Valenciennes, France; nicoblanchard@yahoo.fr; 17 Department of Radiotherapy, Centre Hospitalier Universitaire d’Amiens, 80000 Amiens, France; Coutte.Alexandre@chu-amiens.fr; 18 Department of Radiotherapy, Centre Hospitalier Universitaire de Bordeaux, 33000 Bordeaux, France; charles.dupin@chu-bordeaux.fr; 19 Centre Antoine Lacassagne, Department of Radiotherapy, 06000 Nice, France; shakeel.sumodhee@u-bordeaux.fr; 20 Easy CRF, 8 Rue Lecourtois, 14920 Mathieu, France; v.roth@easy-crf.com; 21 Department of Radiotherapy, Institut de Cancérologie de Lorraine, 54519 Nancy, France; jeanchristophe.faivre@gmail.com; 22 Centre François Baclesse—Centre de Recherche Avancées d’Hadronthérapie Européenne, Department of Radiotherapy, University of Caen Normandie, 14000 Caen, France

**Keywords:** neoplasms/cancers/carcinomas, head and neck, unknown primary, neck dissection, chemoradiotherapy, prognosis

## Abstract

**Simple Summary:**

A retrospective multicentric study of 322 patients with head and neck cancers of unknown primary (HNCUP) was performed testing the impact of neck dissection (ND) extent on nodal relapse, progression-free survival and survival. After 5 years, the incidence of nodal relapse was 13.4%, and progression-free survival (PFS) was 59.1%. In multivariate analysis after adjusting for nodal stage, the risk of nodal relapse or progression was reduced with lymphadenectomy, selective ND or radical/modified ND but survival rates were similar. Patients undergoing lymphadenectomy or ND had significantly better PFS and a lower nodal relapse incidence in the N1 + N2a group, but the improvement was not significant for the N2b or N2 + N3c patients. Severe toxicity rates exceeded 40% with radical ND. In HNCUP, ND improves PFS regardless of nodal stage but fails to improve survival. The magnitude of the benefit of ND did not appear to depend on ND extent and decreased with a more advanced nodal stage.

**Abstract:**

Purpose: Management of head and neck cancers of unknown primary (HNCUP) combines neck dissection (ND) and radiotherapy, with or without chemotherapy. The prognostic value of ND has hardly been studied in HNCUP. Methods: A retrospective multicentric study assessed the impact of ND extent (adenectomy, selective ND, radical/radical-modified ND) on nodal relapse, progression-free survival (PFS) or survival, taking into account nodal stage. Results: 53 patients (16.5%) had no ND, 33 (10.2%) had lymphadenectomy, 116 (36.0%) underwent selective ND and 120 underwent radical/radical-modified ND (37.3%), 15 of which received radical ND (4.7%). With a 34-month median follow-up, the 3-year incidence of nodal relapse was 12.5% and progression-free survival (PFS) 69.1%. In multivariate analysis after adjusting for nodal stage, the risk of nodal relapse or progression was reduced with lymphadenectomy, selective or radical/modified ND, but survival rates were similar. Patients undergoing lymphadenectomy or ND had a better PFS and lowered nodal relapse incidence in the N1 + N2a group, but the improvement was not significant for the N2b or N2 + N3c patients. Severe toxicity rates exceeded 40% with radical ND. Conclusion: In HNCUP, ND improves PFS, regardless of nodal stage. The magnitude of the benefit of ND does not appear to depend on ND extent and decreases with a more advanced nodal stage.

## 1. Introduction

Optimal neck management for patients with head and neck cancer of unknown primary (HNCUP) is still controversial [1]. The American Association of Clinical Oncology (ASCO) guidelines and the US National Comprehensive Cancer Network (NCCN) recommend performing, for small-volume nodal disease, either definitive surgery or radiotherapy (with or without chemotherapy). For higher nodal stages or unresectable lymphadenopathies, they recommend definitive chemoradiotherapy, given the increased morbidity of trimodality therapy, including extensive neck dissection (ND) in advanced nodal disease [2,3]. According to French and British guidelines, current practice is to perform ND as first-line therapy for removable neck lymph nodes followed by adjuvant (chemo) radiotherapy [4,5]. ASCO and NCCN guidelines recommend limiting the treatment of small nodes to a single modality (surgery or radiotherapy). In HNCUP [6,7,8], there is insufficient evidence to prove the effects of ND on nodal control. In the mother publication of the current study by Pflumio et al., of 350 patients, 74.5% had unilateral disease and more than two-thirds of them had bilateral irradiation. The main objective of the study was to address the role of nodal and mucosal irradiation with an original hypothesis that unilateral irradiation would be responsible for 15% more relapses than bilateral irradiation. We showed that the regional control rate and occurrence of mucosal primaries did not differ between patients who had unilateral irradiation and those who had bilateral irradiation, and that severe toxicities were more frequent after bilateral than unilateral irradiation. Moreover, we found that ND improved locoregional control but not survival without addressing the specific role of ND by nodal stage and extent of ND.

To date, ND in combination with adjuvant radiotherapy has not been shown to improve overall survival in HNCUP in comparison with definitive radiotherapy [9,10]. De-intensification at nodal recurrence has evolved toward selective ND and appears to be as effective as modified or radical ND [11]. Moreover, the treatment of HNCUP is evolving toward more selective ND and personalization of radiotherapy volumes [12,13]. For bulky nodes, none of the ASCO, NCCN and British guidelines specifically define nodal resectability criteria.

The aim of our study was to investigate whether ND improved progression-free survival and to what extent it correlated with the extent of ND (selective to radical) and nodal stage in HNCUP patients treated by radiotherapy/chemoradiotherapy.

## 2. Materials and Methods

This multicenter retrospective international study included patients with squamous cell HNCUP diagnosed by nodal fine-needle aspiration (FNA), biopsy, lymphadenectomy or dissection, treated between 2000 and 2015 in 20 health centers from France, Italy and the United States. Exclusion criteria were history of head and neck cancer, history of skin cancer and initial metastatic disease. All the patients underwent nodal and mucosal irradiation (adapted to the site of nodes and their lymphatic drainage from the nasopharynx, oropharynx, hypopharynx, larynx or oral cavity). Radiation therapy was by conformal (3D) or intensity-modulated radiotherapy (IMRT) and was performed with cisplatin when indicated. Patients underwent upfront ND or planned ND after radiotherapy [14]. ND was classified into four types: radical ND, in which spinal nerve (XI), sternocleidomastoid muscle and internal jugular vein were removed; radical-modified ND, in which one or two of these structures were preserved; selective ND, in which all three structures were preserved [14]; as well as lymphadenectomy, where the pathologic node was removed. Additional grouping of cases into N1 + N2a (early-stage and favorable prognosis), N2b (intermediate prognosis) and N2c + N3 (advanced stage and unfavorable prognosis) was performed based on pre-established prognostic groups as in the mother study [13]. It was also helpful, for statistical purposes, to allow sufficiently large groups and powerful estimates. Moreover, involvement of the internal jugular vein, the sternocleidomastoid muscle or cervical nerves could lead to relatively radical neck dissection independently of nodal size. Patients with no information on clinical nodal stage or ND extent were excluded.

Follow-up was performed in accordance with the recommendations of the French Society of Otorhinolaryngology (SFORL) [15] and international NCCN recommendations. Disease evaluations were performed using clinical examination. PET-CT was performed at 12 weeks in case of an equivocal response to radiotherapy, on a case-by-case scenario in the early years of the study and systematically after the publication of the PET-neck study [16]. Local relapses were defined as the emergence of primary mucosal carcinoma of the upper aerodigestive tract and regional relapse as nodal persistence or recurrence. Progression-free survival (PFS) was defined as the time between the date of diagnosis and the date of first recurrence or death, whatever the cause. HNCUP-specific survival was calculated using data from patients who died from HNCUP. For HNCUP-specific survival, we only considered death due to head and neck cancer, and death due to other causes was considered as a competing risk [17,18]. Overall survival (OS) was defined as the time between diagnosis and all-cause death. Severe acute and late toxicities were graded using the National Cancer Institute Common Terminology Criteria for Adverse Events, Version 3.0. Patient, tumor and treatment characteristics, as well as information regarding local, regional and metastatic relapses, were collected as electronic report forms (www.easy-crf.com, accessed on 25 January 2021) based on patient medical records. This study is an ancillary of AMBICUP [13] and was approved by comité de protection des personnes (no. 13/26), Commission nationale de l’informatique et des libertés (CNIL) (National Reference: MMS/SBA/AR148528) and Comité consultatif en matière de recherche dans le domaine de la santé (no. 13.753). In addition, ethical approval was obtained for patient accrual in each participating country according to their own rules.

Statistics: Qualitative variables were described as the frequency and percentage. Quantitative variables were described as their median and interquartile range (IQR), or their mean and their standard deviation. The normality of the distribution was assessed by the Shapiro–Wilk test. Chi-square tests or Fisher exact tests were performed for the comparison of qualitative parameters. ANOVA *t*-tests or Kruskal–Wallis tests were carried out for quantitative parameters according to the normality of the distribution. Local and regional relapses were described according to the Fine and Gray model (FGm), with other relapses and death as competing risks. Metastatic relapse was described with the FGm with death as a competing risk. The Kaplan–Meier method was used to describe PFS and overall survival.

Independent prognostic factors were first investigated by bivariate analysis based on FGm for relapses or HNCUP-specific death and on the Cox proportional hazard model for PFS. Nodal stage, whatever its level of significance, and all parameters with a *p*-value of less than 0.1 in the bivariate analysis were included in a full multivariate model. In order to avoid overfitting, this full model was simplified with backward selection. Nodal stage was included in each model during the model selection procedure to take into account potential selection biases (i.e., the choice of ND extent could be performed according to nodal stage). Only parameters with a *p*-value of less than 0.05 after adjustment for nodal stage were kept in the final reduced multivariate model. Results were expressed as adjusted hazard ratio and 95% confidence interval (95% CI). For each nodal stage separately, the incidence of nodal relapse and PFS were described according to ND extent and were compared using the Gray test and log-rank tests, respectively.

Toxicities were compared across the groups (by separating modified radical and radical ND) with Chi-squared test or Fisher exact test; a sensitivity analysis was performed in the subgroup of patients with bilateral nodal irradiation. All statistical analyses were performed using SAS software (SAS Institute Inc., Cary, NC, USA). *p*-values of less than 0.05 were considered statistically significant.

## 3. Results

Out of the 350 patients of the AMBICUP study who underwent radiotherapy for HNCUP [14], 322 patients with comprehensive information on both clinical nodal stage and ND extent were analyzed. There were 271 males (84.2%), and the mean age was 62.3 +/− 10.3 years. A total of 53 patients (16.5%) had no ND, 33 (10.2%) had lymphadenectomy, 116 (36.0%) underwent selective ND and 120 underwent modified or radical ND (37.3%), 15 of which received radical ND (4.7%). Patient and tumor characteristics are reported in Table 1. ND extent was associated with clinical nodal stage (Table 1, *p* < 0.001). Patients without ND had a higher clinical nodal stage *(p* = 0.003).

A total of 33 patients out of 322 patients underwent neoadjuvant chemotherapy: 24/53 (45.3%) in the group with no ND versus 9 (<10%) in patients undergoing lymphadenectomy or ND (*p* < 0.001). A total of 42 patients (79.2%) not undergoing ND, 25 patients (76.8%) undergoing lymphadenectomy, 64 patients (55.2%) undergoing selective ND and 70 patients (58.8%) undergoing modified radical/ radical ND also had chemotherapy in association with RT (*p* = 0.007). Characteristics of antineoplastic treatments are reported in Table S1.

The median follow-up was 34 months, IQR (17–60). The 3-year overall survival was 78.8%, 95% CI (73.3–83.3).

The 3-year incidence of local (mucosal) relapse was 5.8%, 95% CI (3.5–8.9). ND had no significant impact on mucosal relapse (Table 2). In multivariate analysis, after adjustment on clinical nodal stage, mucosal RT was a protective factor against mucosal relapse (Table 2).

**Figure 1:**
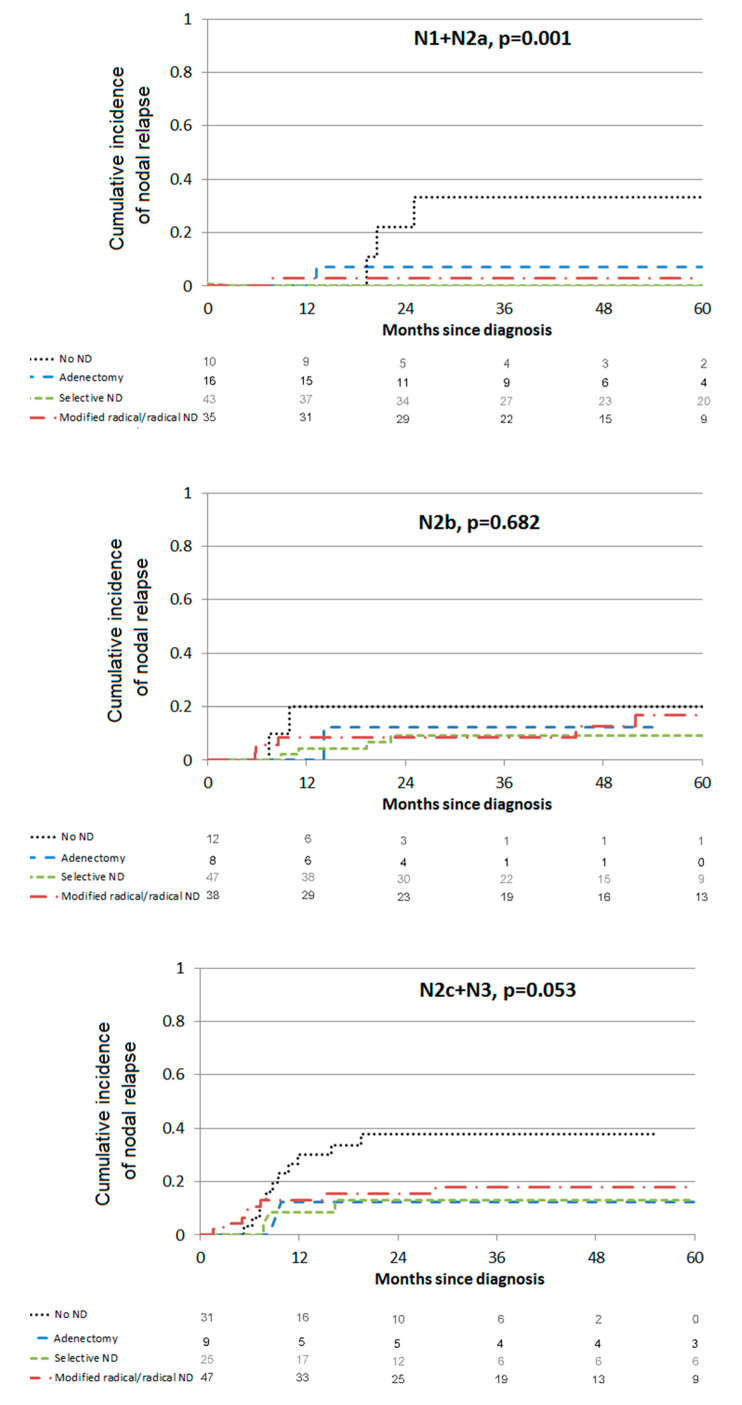
Relapse node.

The 3-year incidence of nodal relapse was 12.5% (95% CI (9.0–16.6)). ND was a protective factor against nodal relapse whatever the extent of ND in bivariate analysis (Table 2). In multivariate analysis, the risk of nodal relapse was reduced with lymphadenectomy (HR = 0.20, 95% CI (0.05; 0.85)), selective ND (HR = 0.20, 95% CI (0.08; 0.51)) or modified/radical ND (HR = 0.40, 95% CI (0.19; 0.83)) whereas N2c-N3 nodes and ≥4-day interruptions of radiotherapy were risk factors for nodal relapse (HR = 2.62, 95% CI (1.06; 6.48) and HR = 3.84, 95% CI (1.71; 8.62), respectively). Impact of ND extent on each nodal stage is presented in Figure 1. Lymphadenectomy or ND significantly decreased the risk of nodal relapse for N1 + N2a patients *(p* = 0.001) but not significantly for N2b or N2 + N3c patients (*p* = 0.682 and *p* = 0.053, respectively).

The 3-year incidence of metastatic relapse was 14.2%, 95% CI (10.5–18.5). ND had no impact on metastatic relapse (Table 2). In multivariate analysis, after adjustment on clinical nodal stage, the incidence of metastatic relapse increased with the largest node diameter (HR = 1.05, 95% CI (1.02–1.08)) whereas initial 18-FDG PET-CT was associated with a lower probability of metastases during follow-up (HR = 0.46, 95% CI (0.24; 0.89)) (Table 2).

**Figure 2:**
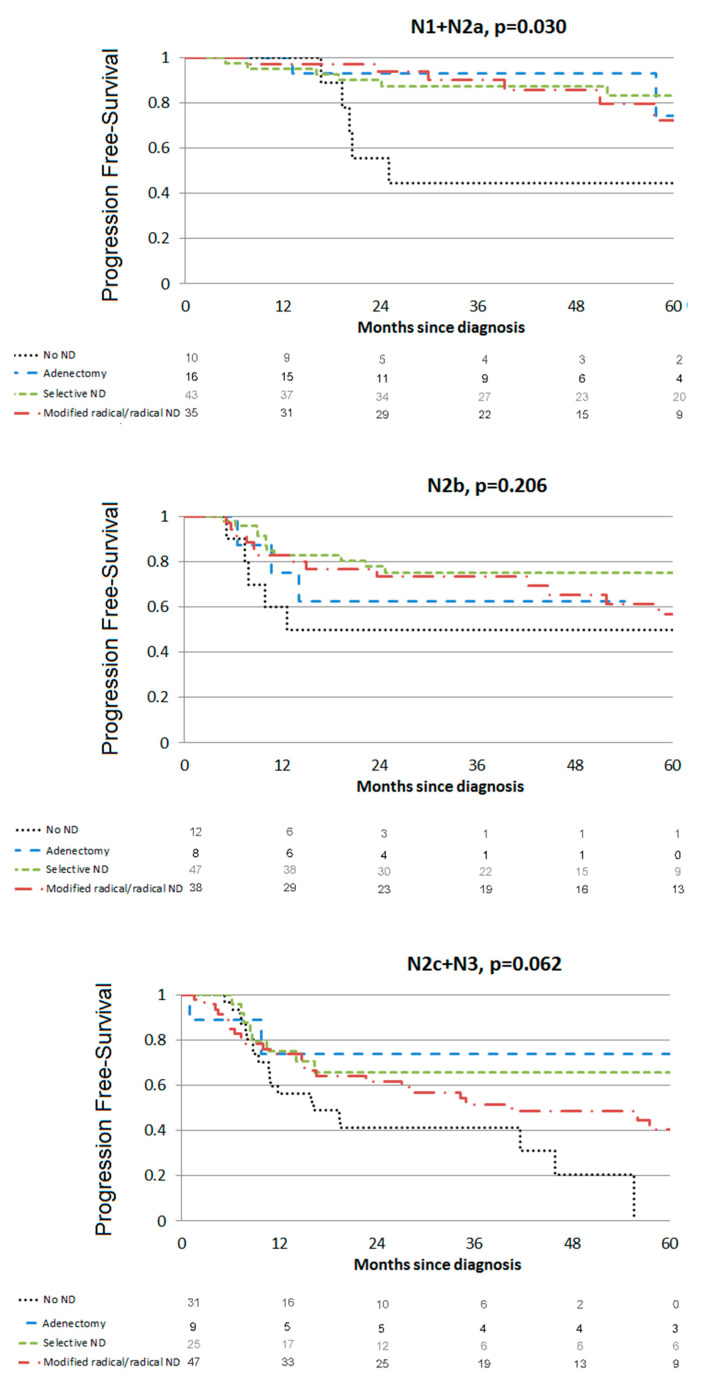
Progression-free survival.

The 3-year PFS was 69.1%, 95% CI (63.3–74.1). In multivariate analysis (Table 3), PFS was poorer in patients with N2b (HR = 1.99, 95% CI (1.18; 3.36)) and N2c-N3 nodes (HR = 3.00, 95% CI (1.83; 4.92)) whereas the PFS was improved with lymphadenectomy (HR=0.29, 95% CI (0.19; 0.67)), selective ND (HR = 0.35, 95% CI (0.21; 0.59)) or modified/radical ND (HR = 0.43, 95% CI (0.27; 0.69)). Prophylactic mucosal radiotherapy was a protective factor (HR = 0.41, 95% CI (0.25; 0.67)) as well as an initial 18-FDG PET-CT (HR = 0.56, 95% CI (0.36; 0.86)). Impact of ND extent for each nodal stage is presented in Figure 2. Patients undergoing lymphadenectomy or ND had significantly better PFS in the N1 + N2a group (*p* = 0.030) but PFS improvement was not significant for N2b or N2 + N3c patients (*p* = 0.206 and *p* = 0.062, respectively).

The 3-year HNCUP-specific death incidence was 16.8%, 95% CI (12.6–21.6). In bivariate analysis, ND, whatever the extent, was a protective factor on HNCUP-specific death (Figure S1), but it did not remain statistically significant in multivariate analysis after adjustment for nodal stage (Table 3). In multivariate analysis, the incidence of HNCUP-specific death was higher for patients with nodes ≥ N2c (HR = 2.55, 95% CI (1.26; 5.15)), with neoadjuvant chemotherapy (HR = 2.45, 95% CI (1.16; 5.18)) and with ≥4-day interruptions of radiotherapy (HR = 3.86, 95% CI (1.75; 8.53)). The incidence of HNCUP-specific death also increased with the largest node diameter (HR = 1.06, 95% CI (1.03; 1.09)) whereas an initial 18-FDG PET-CT was a protector (HR = 0.44, 95% CI (0.24; 0.83)).

Treatment-associated toxicities were more frequent after radical ND than after selective or modified radical ND (Table 4). Toxicity rates were similar between selective ND and no ND. In contrast, radical ND toxicity rates (≥40%) were at least two times higher than with radical-modified ND for late dysphagia and pain. Within the subgroup of the 232 patients with bilateral nodal irradiation, the results were similar (Table S2).

## 4. Discussion

This multicentric retrospective study included 322 patients of a relatively rare subgroup [13,19,20] of head and neck cancer patients with a median follow-up of at least 3 years. This population of patients is very rarely included in clinical trials.

Advanced stage and no neck dissection were associated with poorer regional control in the mother paper [13]. Better local control was reported with mucosal RT, and bilateral nodal irradiation yielded non-significant better nodal and mucosal control rates. The current study, therefore, focused on the impact of ND by nodal stages on oncologic outcomes. ND in combination with radiotherapy improved nodal control and PFS in HNCUP, and this was so for all nodal stages. Advanced nodal presentation was not compensated for by neoadjuvant chemotherapy. Adapted neck management from lymphadenectomy alone, which might be considered as hyper-selective ND, to radical ND, is based on clinical nodal stage and patient-related factors. Interestingly, a benefit of ND on nodal relapse and PFS was achieved with such a strategy. However, patients with advanced nodal stage, i.e., ≥ N2b nodes, did not benefit as much from ND as N1-N2a stages with respect to nodal relapse or PFS.

Lymphadenectomy is not recommended and is usually not considered as ND. However, this is a very intriguing observation of our study and this group (size sufficient for statistics) that we felt it was interesting to show as an exploratory finding that pushes the limits of ND toward even more than hyper-selective ND.

It should be noted that non-resectability and nodal kinetics are uneasily collected items. This is not only true in retrospective databases but also in clinical trials due to the lack of standardization of the definitions. This may reflect some heterogeneity in terms of resectability for the very advanced stages in our study. This might also explain some of the large differences observed between published studies. Demiroz et al. and Colletier et al. found no benefit from ND in locoregional control in comparison with chemoradiation alone, in their small retrospective studies [10,21]. In Colletier’s study, most patients had low nodal stages N1 and N2a (n = 80, 58%), and all nodal relapses occurred in patients who initially had extracapsular spread (n = 12). Other small to medium-size studies have reported better PFS but not better survival [22,23]. Further to our observation, French GETTEC guidelines for resectability criteria have been published to better assess the arguments for ND and the impact of strategies using ND [24].

Patients with N2b-to-N3 nodes had higher rates of HNCUP-specific death, and specific survival was similar in patients with or without ND in our study. In the meta-analysis of Balaker et al., patients undergoing radiotherapy/chemoradiotherapy with ND or without had a 5-year overall survival of 52.4%, compared to 46.6% [9], as in most studies [10,12,19,21,22,23,25,26]. An improvement of survival in patients with ND was shown in two studies only [27,28].

In the mother study [13], severe toxicities were more frequent after bilateral irradiation than unilateral irradiation. In the present study, treatment-related toxicities were more frequent after radical ND than modified radical ND or selective ND and lymphadenectomy. In a sensitivity analysis, we found that treatment-related toxicities were also more frequent for radical ND in the subgroup of patients with more toxicities, i.e., with bilateral radiotherapy. These results suggest that both radical ND and bilateral irradiation induce more toxicities. Altogether, and despite limitations of retrospective studies, our observations may also suggest that adapted ND was efficient across all nodal stages but was less efficient in controlling the nodal and metastatic disease. One intriguing new observation was that lymphadenectomy alone was also beneficial. Although lymphadenectomy is usually considered a diagnostic procedure, this observation suggests that it might be equivalent to hyper-selective ND. This should be investigated further before any therapeutic change is indicated.

Chemotherapy was associated with poor prognosis, suggesting that it did not compensate for advanced nodal stage and the associated risk of metastases. New systemic treatments and additional locoregional treatments, with new approaches such as nanoparticles in large nodes, might be worth being investigated.

In our study, prognostic factors for locoregional relapse, PFS and HNCUP-specific death included an interval of more than 10 weeks between diagnosis and radiotherapy. In head and neck cancers, the longer the delay between initial surgery and adjuvant radiotherapy, the shorter the survival [28,29,30]. The PET-CT prognostic value for HNCUP-specific in our study probably indicated better detection of synchronous metastatic lesions [11,19,29,31,32,33] (and further exclusion of these patients from our studies). It was performed on 20% of patients before 2005 only, 42% in 2005 and 95% after 2006 [13]. PET-CT has also become a standard of care for patients with residual nodes after chemoradiation. Mehanna et al. showed that survival was similar among N2-3 patients who underwent PET-CT-guided surveillance and those who underwent planned ND [33,34,35].

The main limitations of this retrospective study include inaccurate criteria for resectability and inaccurate assessment of the quality of ND (the number of resected nodes was not recorded) [27]. Differences in nodal stage and age were identified between operated and non-operated groups, suggesting unfair comparison. To address this bias, a propensity score analysis was performed (supplementary data). While tonsillectomy was the rule at the time of study, tongue-base biopsies and endoscopically-guided mucosectomies are increasingly used to maximize the chances of non-clinical primary diagnosis, suggesting that some HNCUP may indeed be small oropharyngeal primaries [36,37]. Similarly, HPV and Epstein–Barr virus (EBV) testing have been recommended as systematic in HNCUP only by the latest Union for International Cancer Control (UICC) TNM classification (8th TNM 2017) update. HPV and EBV testing were not feasible due to a lack of specific funding for translational analyses on >300 patients. Of note, p16 staining has only become broadly systematic in France since 2018 for oropharyngeal primaries. This may be different in the USA and in Northern countries [38] where the prevalence of HPV-positive oropharyngeal primaries is higher than in France and Italy. Motz et al. and Schroeder et al. reported that the frequency of HNCUP increased significantly in the past decade and was related to the incidence of HPV [39,40]. Therefore, updates from large HNCUP studies will be useful to compare outcomes with these new practices to those of the current study.

## 5. Conclusions

In patients with HNCUP, ND in combination with radiotherapy improves PFS, regardless of nodal stage but fails to improve specific survival. The magnitude of the benefit of ND did not appear to depend on ND extent and decreased with more advanced nodal stage while toxicity rates exceeded 40% with radical ND in our study. New approaches are warranted to improve nodal control and survival in advanced nodal stages of HNCUP.

## Figures and Tables

**Table 1:** Patient and tumor characteristics.

Neck Dissection Extent			All Patients N = 322	No ND N = 53	Adenectomy N = 33	Selective ND N = 116	Modified Radical/Radical ND N = 120	*p*-Value
**Patients**								
Male			271 (84.2%)	48 (90.6%)	26 (78.8%)	95 (81.9%)	102 (85.0%)	0.414
Female			51 (15.8%)	5 (9.4%)	7 (21.2%)	21 (18.1%)	18 (15.0%)	
Age (years)			61.5; 62.3 +/− 10.3	62.6; 64.4 +/− 11.4	65.6; 64.8 +/− 12.2	61.2; 61.0 +/− 9.5	61.4; 62.1 +/− 9.9	0.330
**Tumors**								
Extension staging, including:								
	Head and neck CT							
		Head and neck MRI	303 (94.1%)	51 (96.3%)	33 (100.0%)	108 (93.1%)	111 (92.5%)	0.353
		Chest-abdomn-pelvic	45 (14%)	6 (11.3%)	0	24 (20.9%)	15 (12.5%)	0.015
		CT	175 (56.6%)	32 (64.0%)	21 (75.0%)	62 (54.9%)	60 (50.9%)	0.082
		18-FDG PET-CT	265 (82.6%)	44 (83.0%)	29 (87.8%)	99 (85.3%)	93 (78.2%)	0.409
Nodal stage:								<0.001
	N1 + N2a		104 (32.3%)	10 (18.9%)	16 (48.5%)	43 (37.1%)	35 (29.2%)	
	N2b		106 (32.9%)	12 (22.6%)	8 (24.2%)	48 (41.4%)	38 (31.7%)	
	N2c + N3		112 (34.8%)	31 (58.5%)	9 (27.3%)	25 (21.6%)	47 (39.2%)	
Diameter of largest cervical node (cm)			4.5; 5.7 +/− 6.1	6; 6.5 +/− 3.6	4; 8.2 +/− 11.2	3.5; 5.6 +/− 7.8	5.0; 4.8 +/− 2.0	<0.001
Extracapsular spread ^a^			209 (71.8%)	25 (75.8%)	21 (70.0%)	74 (64.9%)	89 (78.1%)	0.158
	Conventional squamous cell carcinoma		316 (98.1%)	53 (100%)	33 (100%)	112 (96.6%)	118 (98.3%)	0.354
	Variant of squamous cell carcinoma		6 (1.9%)	0	0	4 (3.5%)	2 (1.7%)	
Histological differentiation ^a^								0.357
	Well differentiated		120 (43.8%)	25 (51.1%)	13 (48.1%)	46 (46%)	39 (37.5%)	
		Keratinizing	85 (31%)	18 (41.9%)	13 (48.1%)	28 (28%)	26 (24.8%)	
		Non-keratinizing	24 (8.8%)	2 (4.6%)	0	12 (12%)	10 (9.5%)	
		NOS	11 (4%)	5 (4.6%)	0	6 (6%)	3 (2.9%)	
	Moderately differentiated		71 (25.9%)	9 (20.9%)	7 (25.9%)	26 (26%)	29 (27.9%)	
		Poorly differentiated	76 (27.7%)	10 (23.4%)	7 (25.9%)	25 (25%)	34 (32.7%)	
		Undifferentiated	7 (2.6%)	2 (4.6%)	0	3 (3%)	2 (1.9%)	
HPV status ^a^								−
	Positive		14 (26.4%)	0	1 (100%)	8 (23.5%)	5 (31.3%)	
	Negative		39 (73.6%)	2 (100%)	0	26 (76.5%)	11 (68.8%)	

Results presented with frequency and percentage (n%) or by median; mean +/− standard deviation. CT, computerized tomography; HPV, human papillomavirus; MRI, magnetic resonance imaging; NOS, not otherwise specified; SCC, squamous cell carcinoma; 18FDG PET, 18fluorodeoxyglucose positron emission tomography. ^a^ Missing data > 10%: extracapsular spread = 31, differentiation = 483, HPV = 292. Totals account for missing data, percentages are calculated with known data only.

**Table 2:** Prognostic factors of local (mucosal), nodal and metastatic relapse of HNCUP in bivariate and multivariate analysis, using the Fine and Gray model for competitive factors.

Prognostic Analysis by Event	Local Relapse				Nodal Relapse				Metastatic Relapse			
	Bivariate Analysis		Multivariate Analysis		Bivariate Analysis		Multivariate Analysis		Bivariate Analysis		Multivariate Analysis	
	HR 95% CI	*p*-Value	HR 95% CI	*p*-Value	HR 95% CI	*p*-Value	HR 95% CI	*p*-Value	HR 95% CI	*p*-Value	HR 95% CI	*p*-Value
**Patients**												
Male gender	2.34 [0.57; 9.59]	0.238			1.74 [0.62; 4.89]	0.292			1.14 [0.49; 2.67]	0.755		
Age at diagnosis	1.04 [0.99; 1.08]	0.091			1.03 [1; 1.06]	0.066			1.04 [1; 1.07]	0.030		
**Tumors**												
Initial imaging 18-FDG PET-CT	0.58 [0.25; 1.32]	0.195			0.92 [0.42; 2.05]	0.844			0.46 [0.24; 0.87]	0.016	0.46 [0.24; 0.89]	0.021
Diameter of largest node (cm)	1.00 [0.96; 1.04]	0.910			1.03 [0.99; 1.06]	0.115			1.05 [1.03; 1.08]	<0.001	1.05 [1.02; 1.08]	<0.001
Nodal staging												
N1 + N2a	1		1		1		1		1		1	
N2b	0.79 [0.28; 2.25]	0.659	0.81 [0.28; 2.30]	0.689	2.13 [0.81; 5.57]	0.124	1.90 [0.71; 5.11]	0.200	1.13 [0.5; 2.52]	0.772	0.76 [0.32; 1.80]	0.536
N2c + N3	1.38 [0.57; 3.35]	0.483	1.41 [0.58; 3.43]	0.448	3.89 [1.6; 9.44]	0.003	2.62 [1.06; 6.48]	0.037	1.87 [0.91; 3.84]	0.089	1.57 [0.75; 3.30]	0.231
Extracapsular spread	0.98 [0.39; 2.47]	0.957			1.6 [0.7;3.66]	0.270			2.74 [1.08; 6.99]	0.034		
**Treatments**												
Interval of >10 weeks between diagnosis and start of RT	2.22 [0.97; 5.05]	0.058			2.04 [1.05; 3.95]	0.034			0.89 [0.49; 1.61]	0.693		
Interruption RT ≥ 4 days	1.9 [0.54; 6.74]	0.320			3.76 [1.58; 8.9]	0.003	3.84 [1.71; 8.62]	0.001	1.94 [0.74; 5.08]	0.177		
Neck dissection												
No ND	1				1		1		1			
Adenectomy	0.28 [0.03; 2.36]	0.243			0.17 [0.04; 0.75]	0.019	0.20 [0.05; 0.85]	0.029	0.60 [0.19; 1.87]	0.381		
Selective ND	0.47 [0.163; 1.38]	0.169			0.17 [0.07; 0.4]	<0.001	0.20 [0.08; 0.51]	<0.001	0.57 [0.27; 1.22]	0.149		
Modified radical/radical ND	0.71 [0.26; 1.92]	0.496			0.36 [0.18; 0.73]	0.005	0.40 [0.19; 0.83]	0.015	0.57 [0.26; 1.22]	0.149		
Mucosal RT	0.32 [0.13; 0.76]	0.010	0.32 [0.13; 0.76)	0.010	0.65 [0.29; 1.46]	0.309			0.52 [0.26; 1.07]	0.076		
Chemotherapy	0.52 [0.24; 1.13]	0.098			1.47 [0.73; 2.93]	0.279			1.67 [0.84; 3.33]	0.142		
Neoadjuvant chemotherapy	1.26 [0.38; 4.19]	0.712			2.74 [1.31; 5.7]	0.007			0.87 [0.32; 2.41]	0.791		
Concomitant chemotherapy	0.5 [0.23; 1.1]	0.085			1.02 [0.54; 1.94]	0.941			1.49 [0.78; 2.86]	0.230		

**Table 3:** Prognostic factors of progression-free survival and HNCUP-specific death in bivariate and multivariate analysis, using the Fine and Gray model for competitive factors.

Prognostic Analysis by Event		Progression-Free Survival				HNCUP-Specific Death			
		Bivariate Analysis		Multivariate Analysis		Bivariate Analysis		Multivariate Analysis	
		HR 95% CI	*p*-Value	HR 95% CI	*p*-Value	HR 95% CI	*p*-Value	HR 95% CI	*p*-Value
**Patients**									
Male gender		1.46 [0.85; 2.51]	0.174			3.82 [1.17;12.45]	0.026		
Age at diagnosis		1.03 [1.01; 1.05]	0.001			1 [0.98;1.03]	0.880		
**Tumors**									
Initial imaging including a 18-FDG PET-CT		0.61 [0.4; 0.92]	0.017	0.56 [0.36; 0.86]	0.008	0.43 [0.25;0.74]	0.002	0.44 [0.24; 0.83]	0.010
Diameter of largest node (cm)		1.03 [1.01;1.06]	0.009			1.06 [1.03;1.08]	<0.001	1.06 [1.03; 1.09]	<.001
Nodal stage									
	N1+N2a	1		1		1		1	
	N2b	1.82 [1.09; 3.06]	0.023	1.99 [1.18; 3.36]	0.010	1.61 [0.77; 3.35]	0.206	0.97 [0.41; 2.29]	0.948
	N2c+N3	3.26 [2.02; 5.25]	<0.001	3.00 [1.83; 4.92]	<0.001	3.65 [1.87; 7.13]	<0.001	2.55 [1.2; 5.15]	0.009
Extracapsular spread		2.13 [1.25; 3.63]	0.006			2.18 [1.04; 4.56]	0.0382		
**Treatments**									
Time lapse between diagnosis and irradiation		1.25 [0.87; 1.8]	0.236			1.03 [0.62; 1.68]	0.923		
Interruption RT ≥ 4 days		1.59 [0.84; 3]	0.155			3.31 [1.58; 6.96]	0.002	3.86 [1.75; 8.53]	<0.001
Neck dissection									
	No ND	1		1		1			
	Lymphadenectomy	0.26 [0.11; 0.58]	0.001	0.29 [0.13;0.67]	<0.001	0.17 [0.04; 0.78]	0.022		
	Selective ND	0.28 [0.17; 0.46]	<0.001	0.35 [0.21; 0.59]	<0.001	0.40 [0.20; 0.80]	0.010		
	Modified radical/radical ND	0.45 [0.29; 0.71]	<0.001	0.43 [0.27; 0.69]	<0.001	0.50 [0.26; 0.96]	0.037		
Mucosal radiotherapy		0.5 [0.31; 0.81]	0.005	0.41 [0.25; 0.67]	<0.001	0.62 [0.32; 1.21]	0.161		
Chemotherapy		1.04 [0.71; 1.52]	0.838			1.11 [0.66; 1.86]	0.690		
Neoadjuvant		2.01 [1.21; 3.33]	0.007			2.42 [1.2; 4.84]	0.013	2.45 [1.16; 5.18]	0.018
Concomitant		0.84 [0.58; 1.21]	0.348			0.84 [0.51; 1.39]	0.502		

**Table 4:** Impact of neck dissection on grade III-IV acute and late toxicities.

Neck Dissection Extent	No ND	Adenectomy	Selective ND	Modified Radical ND	Radical ND	*p*-Value
**Acute toxicities**						
Number of patients	53	33	116	105	15	
Dysphagia	19(35.8%)	6(18.2%)	34 (29.3%)	24 (22.9%)	7 (46.7%)	0.122
Pain	9(17.3%)	4(12.1%)	13 (11.3%)	14 (13.5%)	5 (33.3%)	0.206
**Late toxicities**						
Number of patients	50	31	115	99	15	
Dysphagia	5(10.0%)	0	6 (5.2%)	7 (7.1%)	7 (46.7%)	<0.001
Fibrosis	0	1(3.2%)	10 (8.7%)	3 (3%)	6 (40%)	<0.001
Pain	2(4.0%)	0	2 (1.7%)	1 (1%)	2 (13.3%)	0.068

## Data Availability

The data presented in this study are available on request from the corresponding author.

## Supplementary Materials

The following are available online at https://www.mdpi.com/article/10.3390/cancers13102416/s1, Table S1: Impact of neck dissection on grade III-IV acute and late toxicities in the subgroup of the 232 patients with bilateral nodal irradiation, Table S2: Impact of neck dissection on grade III-IV acute and late toxicities in the subgroup of the 232 patients with bilateral nodal irradiation. Figure S1: Incidence of HNCUP specific death according to the extent of ND.
