# Supplementary material for: Impact of Neck Dissection in Head and Neck Squamous Cell Carcinomas of Unknown Primary
Source: Cancers (Basel). 2021 May 17;13(10):2416. doi: 10.3390/cancers13102416 (PMC8156697; doi:10.3390/cancers13102416)
Supplement: Supplementary file 1 [file cancers-13-02416-s001.zip › cancers-1200261-supplementary.pdf]

Supplementary Table 1: Characteristics of irradiation and other antineoplastic treatments.

	All patients N=322	No ND N=53	Adenectomy N=33	Selective ND N=116	Modified radical/ radical ND N=120	p-value
**Radiotherapy**						
**Nodal irradiation**	**322(100%)**	**53(100%)**	**33(100%)**	**116 (100%)**	**120 (100%)**	-
Laterality of radiotherapy (N=287)[a]						<.001
Unilateral	55(19.2%)	10(21.3%)	14(46.7%)	17(16.2%)	14(13.3%)	
Bilateral	232(80.8%)	37(78.7%)	16(53.3%)	88(83.8%)	91(86.7%)	
Duration of radiotherapy (days)	49;48+/-11	53 ;53+/-8	49 ;	49+/-13	47;46+/-10.5	<.001
Dose (Gray Gy)	66;64+/-7	70 ;68+/6-	66 ;66+/-7	66;63+/-7.5	66;63+/-6	<.001
Interval of >10 weeks between diagnosis and irradiation	168 (52.2%)	35(66.0%)	14(42.4%)	61 (52.6%)	58 (48.3%)	0.109
**Mucosal irradiation**	**281(87.8%)**	**47(90.4%)**	**27(81.2%)**	**103 (88.8%)**	**104 (87.4%)**	0.670
Nasopharynx	209 (67.4%)	30(60.0%)	23(76.7%)	75 (66.4%)	81 (69.2%)	0.448
Oropharynx	271 (85.2%)	44(86.3%)	26(81.2%)	100 (86.2%)	101 (84.9%)	0.909
Hypopharynx	243 (76.9%)	40(78.4%)	24(75.0%)	84 (73.7%)	95 (79.8%)	0.712
Larynx	207 (65.9%)	35(68.6%)	14(43.7%)	70 (62%)	88 (74.6%)	0.008
Oral cavity	72 (23.8%)	15(31.2%)	5(16.7%)	33 (30%)	19 (16.5%)	0.046
Interruption ≥ 4 days	21 (6.5%)	4(7.5%)	3(9.1%)	6 (5.2%)	8 (6.7%)	0.856
**Chemotherapy**	**210 (65.4%)**	**46(86.8%)**	**26(78.8%)**	**65 (56.0%)**	**73 (61.3%)**	<.001
Neoadjuvant	33 (10.3%)	24(45.3%)	3(9.1%)	3 (2.6%)	3 (2.5%)	<.001
Concomitant	201 (62.6%)	42(79.2%)	25(76.8%)	64 (55.2%)	70 (58.8%)	0.007

35 patients for whom laterality of radiotherapy was missing.

Results presented as frequency and percentage (n%) or by median; mean +/- standard deviation.

Supplementary Table 2: Impact of neck dissection on grade III-IV acute and late toxicities in the subgroup of the 232 patients with bilateral nodal irradiation.

	**No ND**	**Lymphadenectomy**	**Selective ND**	**Modified radical ND**	**Radical ND**	**p-value**
**Acute toxicities**						
Number of patients	*37*	*16*	*88*	*78*	*13*	
Dysphagia	15(40.5%)	3(18.7%)	29(32.9%)	19(24.4%)	7 (53.8%)	0.111
Pain	8(21.6%)	1(6.2%)	13(14.9%)	14(17.9%)	5(38.5%)	0.190
**Late toxicities**						
Number of patients	*37*	*15*	*88*	*74*	*13*	
Dysphagia	5(13.5%)	0	6 (6.8%)	5 (6.8%)	6 (46.1%)	0.002
Fibrosis	0	0	8(9.1%)	3(4.0%)	5(38.5%)	0.001
Pain	2(5.4%)	0	2 (2.3%)	1 (1.3%)	2 (15.4%)	0.109

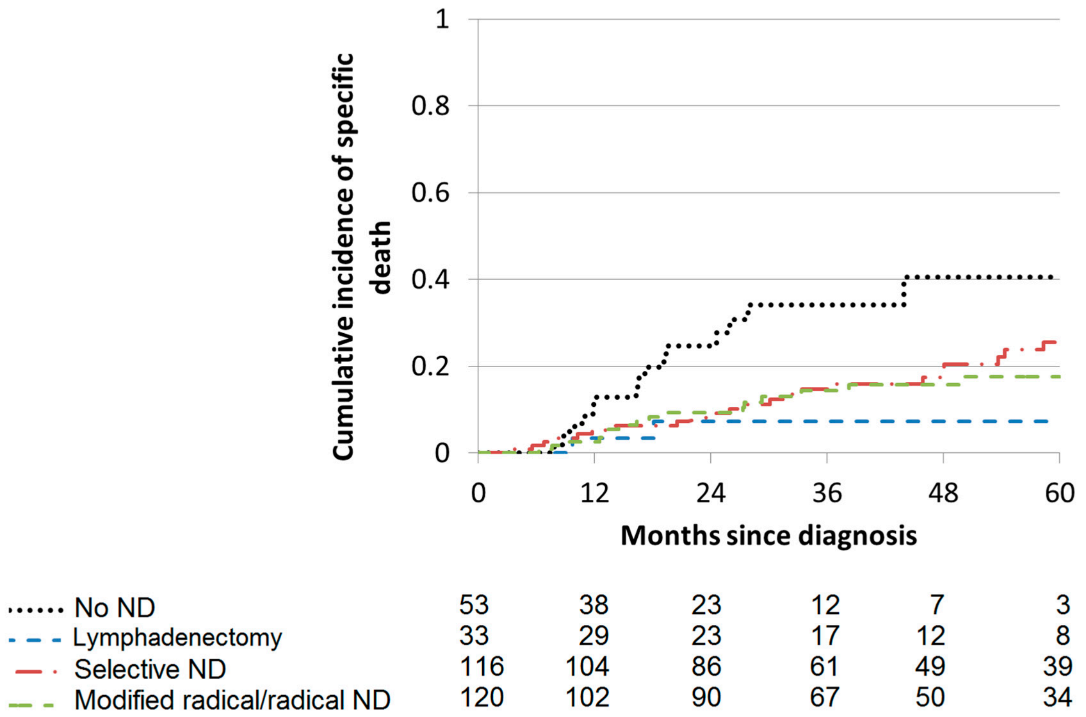

Supplementary Figure 1: Incidence of HNCUP specific death according to extent of ND.
